# The Foreign Journals: Surgery

**Published:** 1839-01

**Authors:** 


### SURGERY.

*On the Removal of the Bones of the Face.* By Professor J. F. Dieffenbach.

The attention which modern surgeons have given to remedy or remove the diseases which attack the face has induced Dr. Dieffenbach to give his experience on the subject, in the form of a report of a number of cases in which he had successfully removed portions or the whole of the upper and lower jaw.

The superior maxilla was first removed by Professor Lizars, of Edinburgh. Since his time numerous surgeons, both in Great Britain and on the continent, have performed this operation. In a paper by Mr. Liston, published in the 20th volume of the Medico-Chirurgical Transactions, on this subject, an account is given of those diseases which affect the upper and lower maxilla; and those are distinguished in which an operation may be ventured upon with hopes of success. The diseases are the following: 1. Parulis, or spina ventosa; swellings of an acute or chronic nature, from which there is usually a purulent secretion. 2. Epulis; a solid growth from, and of the consistence of gum. 3. A fungous vascular growth from the apex of the fangs of teeth, which increases gradually, bleeds easily, and beneath which the osseous tissue becomes changed into a soft lardaceous brain-like matter. 4. Osteo sarcoma; a soft, ragged, foul, ulcerated, fungous growth, commencing usually amongst the bones, which it gradually displaces; sometimes bleeding, and always malignant. 5. Tumours of erectile tissue, usually occupying the antrum. 6. Simple fibrous tumours, having a botryoidal form, and slow in growth, attaining a great size, and not malignant.

The parulis and epulis are usually slight affections, involving only small portions of the alveolar process, and which may be easily removed. The fibrous tumours may always be safely removed: they are slow in growth, and not of a malignant character. The other diseases should only be interfered with at an early period, when the neighbouring glands are not affected, and when the whole disease, together with the surrounding healthy parts, may be removed.

Of the cases reported by Dieffenbach, twelve involved only portions of the jaw, and were usually of the nature of the parulis or epulis. Three cases, in which the whole of one of the superior maxillary bones was removed, would appear, from the slowness of their growth, and the size they had attained, to have been of the nature of the fibrous or botryoidal tumours of Mr. Liston. One case of the lardaceous or brain-like affection of the bone was submitted to five different operations before the disease was extirpated. M. Dieffenbach calls one case Fungus malonodes, and which he successfully treated by removal of the superior maxillary bone. A fibrous tumour of the lower jaw was also removed successfully, together with a portion of the bone.

All the cases reported by Dieffenbach had a favorable termination.

[After the first report of fifteen cases of removal of the upper maxillary bone, and of which eleven proved fatal, this operation was unfavorably thought of. But if we keep in mind the rules of diagnosis laid down by Mr. Liston, and consider the termination of the various cases which have been operated on, we may consider this as one of the most successful operations in surgery. Mr. Liston has removed the superior maxilla seven times, with only one case of failure. Dr. Dieffenbach has had five operations, and no failure. Gensoul has removed the superior maxilla four times, with one case of failure; and then the affection was of a malignant nature. Regnoli had one successful case, and one failure. Of the eleven cases operated on by Messrs. Lizars, Syme, Robert, Scott, Earle, Guthrie, and Hetling, only one case was completely successful; but no regard was paid to the malignant or simple nature of the disease.

In the performing of the operation of removing the upper jaw, Dr. Dieffenbach insists much on the necessity of dividing the skin as much as possible in the middle line. This appears objectionable, inasmuch as it would cause much more deformity of the nose. Mr. Liston carries the incision always round the ala of the nose, and thus this organ retains its natural appearance.]

*Hamburg Zeitschrift f. d. g. Med. Feb.* 1838.

### *Influence of Temperature on the Cicatrization of Wounds.* By MM. BRESCHET and GUYOT.

AT the sitting of the Academy of Sciences on the 2d July, a joint memoir was presented by these gentlemen, containing the result of some experiments made at the Hôtel Dieu, on the cicatrization of wounds under the influence of an elevated temperature. For this purpose a hot-air bath was contrived, in which a diseased limb might be exposed to a dry temperature of 36 centigrade (97° F.) A piece of glass inserted in the top of the apparatus enables the progress of cure to be watched without disturbing the limb. Two cases are detailed, in which, immediately after amputation, the stumps were placed in this apparatus. In the first case, in which amputation was performed for scrofulous disease of the femur, no other dressing was applied than five very narrow strips of emplast. plumbi, to approximate the skin without covering the wound, and a roller round the thigh. These slight dressings were removed on the fourth day, when union had taken place except at the inferior portion: here there was a trifling suppuration, which ceased on the fourteenth day after the removal of the ligatures. The superior three fourths of the wound remained dry during the whole process of cure. On the fifteenth day there remained a wound from fifteen to twenty lines in length by only one in breadth. The constitutional state of the patient was most satisfactory throughout. In the second case, that of a man aged sixty-one, amputation was performed on account of suppuration in the ankle-joint. There had been a good deal of constitutional irritation, and the stump was flabby and infiltrated; the tibial artery was ossified. Here union by the first intention was not attempted. Four strips of adhesive plaster were applied, which left a space of eighteen lines between the lips of the wound. For the first five days the wound was covered with a brown scab; on the sixth day suppuration commenced; the appearance of the wound was very favorable; the constitutional symptoms were immediately much ameliorated. This case is not yet terminated; but cicatrization is commencing, and everything leads to expect a favorable result.

*Gazette des Hôpitaux. July* 7, 1838.

---

### *Curious Case of a Spike of Oat introduced into the Bronchi, and expelled through an Opening in the Walls of the Chest.* By M. STANSKI.

A YOUNG woman, aged twenty, was brought into the Hôpital Cochin, July 16, 1834. It appeared that she had for some time laboured under consumptive symptoms, and that, a fortnight before her admission, she accidentally swallowed (as she thought) an ear of wild oats, in the act of speaking with it in her mouth: she could not tell which end of the spike went down first. She was immediately seized with a violent fit of choking; which, however, soon went off, and was succeeded by continual convulsive coughing. After two or three days she was attacked with pneumonia of the right lung; and, two or three days subsequently, a sudden fit of coughing was followed by an abundant purulent expectoration of very fœtid matter, which continued till the time of her admission into the hospital. Upon examination, an abscess was detected in the right lumbar region. This abscess was opened by means of caustic, and twelve ounces of purulent matter, resembling that expectorated, was evacuated. Considerable relief of all the symptoms followed this discharge; but cavernous and amphoric respiration, with pectoriloquy, could be heard at the base of the right lung. Subsequently another abscess formed between the ribs, a little below the inferior angle of the scapula; which, after a month, was also opened with caustic. A seton was introduced into the lower opening of the first abscess, and brought out at the newly-made one: by drawing the cotton upwards, the oat-ear was entangled and brought out, broken into two pieces, which together measured three inches in length. After the removal of the foreign body, the wound kept discharging for a considerable time, but ultimately healed, and all the symptoms, both general and auscultatory, decreased; but the frequent pulse and night-sweats remained, and the patient died from phthisis seven months after coming into the hospital.

On examination after death, extensive tubercular disease of the summit of both lungs was found. The lower and back part of the right lung was closely adherent to the ribs by a hard and almost cartilaginous substance: this was continuous with a mass of dense cellular tissue, which descended beneath the pleura costalis, and passed out of the chest between the eleventh and twelfth ribs, close to the outer edge of the sacro-lumbalis muscle, and was continued beneath the lumbar fascia, which was separated from the muscles for the space of one and a half or two square inches. The cavity thus formed was lined by a soft, unorganized, false membrane: it did not communicate with the chest, but led by a small fistulous passage to one of the external openings; the ligamentous adhesion was closely applied to the pleura covering the lung, and at that point a small cylindrical canal was found, which communicated with the largest bronchial tube of the inferior lobe of the lung: the canal itself seemed formed by a dilated bronchus. The surrounding substance of the lungs was friable, and of a grayish brown colour; but almost free from tubercles, only one being found.

M. Stanski says that he has found as many as twenty cases similar to the preceding, reported in different works, and the effects and symptoms of the accident were always the same. The spikes of different grasses put into the mouth have got into the respiratory tubes, while the patient has been speaking, laughing, or coughing. To get in this way into the trachea, the lower extremity of the spike must have been introduced first, otherwise the spikelets would have diverged, and obstructed its passage. It rapidly finds its way into some bronchial tubes, where it excites irritation and suppuration, and eventually reaches the surface of the body, and is discharged along with the matter which surrounds it. Any operation for the removal of the ear is to be condemned: it traverses the trachea with the greatest rapidity during the act of inspiration, as is proved by the short duration of the fit of suffocation; and, in all the cases that M. Stanski has found, the process by which the foreign body has been got rid of has never produced death, except when complicated with other disease, as in the present case, where the patient evidently died of phthisis after recovering from the accidental affection, and in consequence of tubercles existing in the summit of the lungs previously to the occurrence of the accident. *Gazette Médicale de Paris. July* 1, 1837.

---

*New Theory and Treatment of Erysipelas.* By M. Blandin, Surgeon to the Hôtel Dieu, Paris.

Erysipelas may be divided into two varieties, according to the causes which give rise to it. These may be either external or internal; as a wound or injury, or a disordered state of the constitution generally; and the disease will vary considerably in its characters as the one or the other cause may occasion it. According to M. Blandin's theory, that variety which is excited by external injury is *at first* a local affection, and afterwards tends to diffuse itself generally; the fluids, which are altered by the diseased actions going on in the part, having a concentric course, and thus spread themselves through the whole system and excite violent reaction. Erysipelas, on the contrary, arising from an internal cause is at first generally diffused, and has a tendency to become localized; nature making an effort to determine the disturbing influence towards a single point.

With regard to the anatomical nature or proximate cause of erysipelas, M. B. considers that it consists in acute inflammation of the minute lymphatic vessels of the skin, which are first affected, and inflammation of the substance of the skin itself afterwards follows. [This idea of the absorbents being inflamed in erysipelas is not new, though M. B. is the first who has extensively applied it, in numerous cases, both to pathology and therapeutics: this same theory has been entertained by several authors; among others, MM. Ribes, Dance, and Chomel.] The degree of inflammation between the lymphatic capillaries and the substance of the skin is not always in equal proportion. In erysipelas from internal causes, it is the latter which predominates; in the traumatic variety, the absorbents are principally affected: and this causes the difference in the seriousness of the affections; for according to M. B., physicians generally find erysipelas a trifling disorder, while surgeons regard it as a most serious disease.

M. Blandin's treatment is founded on the principle that the preexisting and predominant affection is inflammation of the lymphatics, and that, when this is checked, there only remains simple inflammation of the integuments. As the disease is propagated towards those lymphatic ganglions which are situated most centrically, or nearest to the trunk, it is here that we should commence the treatment, which consists in successive applications of leeches over the absorbent glands, and not on the erysipelatous surface of the skin itself; as in the latter case they only weaken the patient. This plan is principally applicable to the traumatic variety, when situated in the extremities; but he considers that it may be also employed with advantage when the skin of the trunk is affected, and when the disease arises from internal causes.

M. B. has applied his plan of treatment to a great number of cases, and informs us that, during two years that he has employed it, he has scarcely lost a single patient; but he ought to publish the exact number of instances in which he has tried it, with the particulars of the different cases: this would render his statements much more valuable.

*Journal des Connaissances Médico-chirurgicales. July,* 1837.

---

*New Mode of tying Polypi of the Pharynx.* By Dr. FELIX HATIN.

THE tumour in this case was fixed by a large pedicle to the base of the cranium, and descended vertically into the pharynx, which it filled, reaching to the uvula; the soft palate was pressed downwards and forwards by it; the passage of the breath through the nostril was entirely impeded; the voice was what is improperly called nasal, and deglutition difficult. The operation was performed with a quickness and facility which M. Lisfranc admitted was surprising; and Dr. Hatin thus explained the method he adopted: In the ordinary method, an elastic sound must first be passed through one of the nostrils, and the posterior extremity, being brought forward into the mouth, is to be attached to the two ends of a ligature, in which a noose has been previously made; the sound is then to be drawn back, and the extremities of the ligature pulled into the nostril, while the noose passes towards the back of the mouth. The great difficulty is to preserve the loop of the proper dimensions, and to get it over the extremity of the tumour; a thing by no means easily accomplished, in consequence of the depth of the situation in which the polypus is fixed, and the convulsive movements caused by the fingers of the operator in the throat of the patient.

To overcome these difficulties, Dr. Hatin has invented an instrument (called *porte-ligature pharyngien*), the construction of which is very simple. It consists of a flat metallic blade, curved at one extremity, which is to be introduced into the mouth through the noose in the ligature, and placed under the polypus, so that the ligature may be conducted over it. For the purpose of widening the curved extremity, so as to adapt it to the width of the tumour, two other narrow and flat metallic blades are fixed on the upper surface of the instrument, each of which is divided by a hinge. At the straight extremity of the instrument which is taken hold of, these two pieces diverge from each other, and are connected by a screw: when this is turned, the blades at the proximal end are brought nearer together, and diverge at the curved end (beneath the polypus), which is thus widened. To render the instrument complete, a stilette fixed to a spring, with two little hooks at its extremity, is placed in a groove at the lower side of the instrument; the ligature is fixed to the hooks, and, by pressing on a button at the end of the stilette, the noose is conducted at once to the bottom of the tumour, over which, by pulling the threads through the noose, it may easily be made to slip. It is difficult to explain the mechanism of the instrument by a description; but the above will serve to give some idea of a contrivance by which a most difficult operation is rendered perfectly easy.

[From the difficulties attending its application, the ligature is hardly ever applied to the removal of polypi in the nose or fauces in this country. The instrument of Dr. Hatin seems capable of removing this difficulty in a great measure in tumours in the latter situation; but still one imperfect part of the operation must be the difficulty of drawing the noose tight round the base of the tumour, after getting it over the extremity: this can be but imperfectly accomplished by pulling the extremities of the ligature through the nose.]

*Revue Méd. fran. et étran. Sept.* 1838.

---

*Recovery from a Wound of the Ascending Aorta.* By Dr. Heil.

Joseph Hoffman, æt. thirty-two, a Bavarian soldier, while engaged in a drunken quarrel in the year 1812, received a stab in the left side. The weapon which was a common table-knife penetrated the thorax between the fifth and sixth ribs. The left lung was wounded, and the stab was followed by copious hemorrhage. He remained some time exposed to the cold air, and when the doctor saw him, which was about an hour after the receipt of the wound he was lying still bleeding but apparently lifeless. He was removed to the hospital, and the edges of the wound brought together by plaster, although blood still continued to ooze. After having remained some hours unconscious he came to himself, but was then amaurotic, probably from the abundant depletion which he had undergone. In about four months the external wound had entirely healed, but the amaurosis in spite of all the remedies employed still continued; and in this state he left the hospital. He led it appears an irregular life, and some months afterwards was again brought into the hospital labouring under an attack of pneumonia, of which he died in the year 1813. On examining his body, the cicatrix of the wound in the chest was plainly visible: in laying open the cavity, it was found that the correspondent part of the lung was adherent to the pleura, and that the cicatrix extended through the whole substance of the lung to the back part of the organ, where the weapon must have passed out. From this point the knife appeared to have extended upwards towards the ascending aorta; and in this vessel there was an opening with an irregular border about a quarter of an inch in length, which was occupied by a firm coagulum of blood, (thrombus.) On laying open the vessel the weapon was found to have penetrated completely into its cavity. There was a rounded cicatrix on the inside completely closed up as on the outside. The extraordinary cure had probably taken place from a coagulum having formed during the syncope, into which the man was plunged after receiving the wound, aided by the cold to which he was exposed.

[The description of the cicatrix in the aorta is not very clearly given; but the case is altogether very remarkable, as it shows that wounds of this important vessel are not always *absolutely* mortal, as they are laid down in some systems of classification.] *Henke's Zeitschrift.* 1837-8.

---

*Treatment of Varicose Veins.* By M. Velpeau.

M. Velpeau continues to treat varices, and to cure them radically, by passing a pin beneath the varicose vein at a slight distance from the swelling of the vessel. Then, by means of a thread wound over each protruding extremity of the pin, he compresses the vessel and the tissues interposed, so as to stop the circulation in the vein. This procedure, causing but little pain, is said almost always to succeed in the hands of M. Velpeau. It very rarely gives rise to phlebitis, and, when this does happen, it is seldom in any great degree. [We extract this, not because of its novelty, but because it is not a practice generally known, and we are aware of its successful application. It appears to hold out a hope of permanent relief in a most troublesome affection.] *Gazette des Hôpitaux.* No. 53. 1838.

---

*On the immoveable Fracture-bandage.* By Dr. Fricke.

Dr. Fricke has given reports of sixteen cases of fracture successfully treated by means of Seutin's starch-bandage. He applies it by first rolling the limb with a common broad bandage, over which a layer of starch is placed. On this two pasteboard splints, the length and shape of the fractured limb, and nearly encircling it, are applied wet; then a layer of starch, and over this a roller, after each turn of which the starch is applied; the whole being covered with paper, to prevent its sticking. Fricke has usually found the application of this bandage successful.

The following are some of the advantages attending the use of this bandage:—1. The materials are readily procurable. 2. The apparatus lies close to the inequalities of the extremity. 3. Any part of it can be renewed without disturbing the other parts. 4. It is light, and does not prevent the use of the extremity. 5. The limb is so firmly supported, that in two days the patient can leave his bed and walk about.

If the lower limb be fractured, it should be supported by means of a sling round the neck. In applying this bandage, Fricke recommends the surgeon to wait for the subsidence of swelling and inflammation. He does not think, with Seutin and Velpeau, that the patient might even undertake a journey immediately after the injury when the bandage is dry.

*Hamburg Zeitschrift f. d. g. Med. April,* 1838.

*Deglutition of a large Silver Coin.*

An Austrian soldier offered to swallow a coin nearly as large as a crown-piece, on the condition that it should thereupon belong to him: but, on his attempting to do so, it remained in the œsophagus. Emetics were given in vain, and all mechanical attempts failed. Eventually it descended to the stomach. It was necessary to bleed him twice, to remove the inflammation which had been caused by the attempts to remove the coin. The bowels continued costive during four days, notwithstanding the use of laxatives and injections. On the sixth day the money reappeared, whilst the patient was evacuating his bowels.

*Journal des Connaissances Médico-chirurgicales. Jan.* 1838.

